# Should we deny ICU admission to the elderly? Ethical considerations in times of COVID-19

**DOI:** 10.1186/s13054-020-03050-x

**Published:** 2020-06-09

**Authors:** Lenneke E. M. Haas, Dylan W. de Lange, Diederik van Dijk, Johannes J. M. van Delden

**Affiliations:** 1 Department of Intensive Care, Diakonessenhuis, PO box 80250, 3508 TG Utrecht, the Netherlands; 2 Department of Intensive Care Medicine and Dutch Poisons Information Center, University Medical Center Utrecht, Heidelberglaan 100, 3584 CX Utrecht, the Netherlands; 3 Department of Intensive Care Medicine, University Medical Center Utrecht, Heidelberglaan 100, 3584 CX Utrecht, the Netherlands; 4 Department of Medical Humanities, University Medical Center, University Utrecht, Heidelberglaan 100, 3584 CX Utrecht, the Netherlands

**Keywords:** ICU, Critical care, Ethics, Triage, Age, Elderly, COVID-19

## Introduction

The SARS-CoV-2 (COVID-19) pandemic leads to severe shortages of intensive care unit (ICU) facilities in many countries. Although most people appear to be asymptomatic, some reports suggest that 5 to 25% of infected people require hospitalization and 2–4% require mechanical ventilation [1]. This strains many ICUs beyond their maximum capacity. National critical care societies have adopted protocols to increase their beds up to 200% or more. However, although a lot of effort can be done to increase the ICU capacity, demand may still outpace the supply. As a consequence, a scenario can arise in which not every patient who needs ICU treatment can be admitted, and difficult decisions about allocation of ICU beds need to be made [2–4]. In this article, we discuss the use of age as a criterion for ICU treatment in times of scarce ICU capacity by contrasting it with deciding under normal conditions.

## Deciding about ICU treatment under normal conditions

Medical treatment has to be justified by serving the wellbeing of the patient, and it should be aligned with the wishes of the patient. The burden of an ICU treatment has to be carefully balanced against the estimated chance of recovery. This chance of recovery is affected by age and many other factors like the admission diagnosis, severity of organ failure, comorbidities, frailty, and pre-admission performance status [5]. Sometimes, ICU admission might be more appropriate for a fit 90-year-old patient than for a vulnerable 65-year-old patient.

Elderly patients (defined as 70 years and older) have a higher risk of death and of functional decline than younger patients. However, the majority of them survives, and in addition, several studies have demonstrated that elderly ICU survivors might accept their disabilities and accommodate to a degree of physical disability quite well, consider their quality of life to be good or satisfactory, and report good emotional and social well-being after hospital discharge [6].

The carefully balancing of pros and cons of ICU treatment should be done before ICU admission (as Advance Care Planning) but also during a (prolonged) ICU admission.

What is common to all decisions on starting, continuing, or foregoing life support is that they should be justified by the autonomous wish of the patient and the benefit of treatment for that unique patient. Age may play a role in these decisions in several ways. It is proxy for the medical condition of the patient, and advanced age is clearly a factor that should be weighed together with other risk factors for a poor outcome of ICU treatment. Elderly patients themselves may also have the feeling that they have lived life to its full and that therefore life-sustaining treatments should not be applied in their own case. There is, however, no valid reason to limit ICU admissions to those under a specific age.

## Outcomes of elderly ICU patients with COVID-19

Elderly patients admitted to the ICU with COVID-19 are at increased risk of death [7, 8]. Although we need more robust data about short-and long-term outcomes of elderly patients admitted to the ICU because of COVID-19, the mortality rates reported up to now are 40 to 80% [7, 9]. These numbers will even become higher, since at the time of reporting a substantial portion of the patients was still in the ICU and the follow-up was short.

## Using age as a selection criterion in time of scarcity

In circumstances of a pandemic, not only the autonomy of the patient and proportionality of treatment, but also shortage of resources may play a role in decisions about ICU treatment. Emanuel and colleagues proposed to use a utilitarian framework [10]. This strategy aims to maximize the benefits for the largest number of people and prioritize care based on the (estimated) greatest advantage of ICU treatment, the so called incremental probability of survival. According to this approach, for instance, parents of young children should be prioritized, then parents of teenagers, middle-aged people, then elderly. Chances of survival rates after ICU admission decrease with increasing age, making age an important factor in this utilitarian approach.

The use of age as a selection criterion in case of scarcity can also be justified by pointing at the “fair innings” that a patient has had, meaning that older patients have already had their opportunity to reach a certain “mature” age, which has given them a fair equality of opportunity. The idea is that everyone should have an equal opportunity to lead a life of a certain duration. While there is no hard and fast rule for what is an unfulfilled life age for a person, most policies distributing lifesaving resources look to those under 18 as gaining priority while those in their 80s and beyond, who have had a chance to experience life and flourish as human being, receive lower priority. We submit that this strategy does not amount to age discrimination as all people are treated alike: when they become older, their claim on life-sustaining treatment decreases.

## Conclusion

In this article, we discussed two ways of using age in the triage of ICU admission. Under normal circumstances, age should be weighed as a risk factor for poor outcome. Together with other risk factors, it may lead to the shared decision to forego ICU treatment. It cannot be justified to withhold ICU admission for all patients above a certain age. In times of scarcity, however, we believe it is justified to prioritize the younger patients, in order to maximize the benefits for the largest number of people, and because of the fair innings that an elderly patient has already had.

## Data Availability

Not applicable.

## Abbreviation

ICU: Intensive care unit

## References

[1] Guan W-J, Ni Z-Y, Hu Y, Liang W-H, Ou C-Q, He J-X (2020). Clinical characteristics of coronavirus disease 2019 in China. N Engl J Med.

[2] Phua J, Weng L, Ling L, Egi M, Lim C-M, Divatia JV (2020). Intensive care management of coronavirus disease 2019 (COVID-19): challenges and recommendations. Lancet Respir Med.

[3] Rosenbaum L. Facing Covid-19 in Italy - ethics, logistics, and therapeutics on the epidemic’s front line. N Engl J Med. 2020;382(20):1873–5. 10.1056/NEJMp2005492. Epub 2020 Mar 18. 10.1056/NEJMp2005492 32187459

[4] Christian MD, Sprung CL, King MA, Dichter JR, Kissoon N, Devereaux AV (2014). Triage: care of the critically ill and injured during pandemics and disasters: CHEST consensus statement. Chest..

[5] Guidet B, de Lange DW, Boumendil A, Leaver S, Watson X, Boulanger C (2020). The contribution of frailty, cognition, activity of daily life and comorbidities on outcome in acutely admitted patients over 80 years in European ICUs: the VIP2 study. Intensive Care Med.

[6] Kaarlola A, Tallgren M, Pettila V (2006). Long-term survival, quality of life, and quality-adjusted life-years among critically ill elderly patients. Crit Care Med.

[7] Grasselli G, Zangrillo A, Zanella A, Antonelli M, Cabrini L, Castelli A, et al. Baseline characteristics and outcomes of 1591 patients infected with SARS-CoV-2 admitted to ICUs of the Lombardy region, Italy. JAMA. 2020;323(16):1574–81. 10.1001/jama.2020.5394. Online ahead of print. 10.1001/jama.2020.5394 PMC7136855 32250385

[8] Yang X, Yu Y, Xu J, Shu H, Xia J, Liu H (2020). Clinical course and outcomes of critically ill patients with SARS-CoV-2 pneumonia in Wuhan, China: a single-centered, retrospective, observational study. Lancet Respir Med.

[9] ICNARC report on COVID-19 in critical care. 08 May 2020. ICNARC Case Mix Programme Database. https://www.icnarc.org/Our-Audit/Audits/Cmp/Reports. Accessed 19 Apr 2020.

[10] Emanuel EJ, Persad G, Upshur R, Thome B, Parker M, Glickman A, Zhang C, Boyle C, Smith M, Phillips JP.Emanuel EJ, et al. N Engl J Med. 2020;382(21):2049-55. 10.1056/NEJMsb2005114. PMID: 32202722. 10.1056/NEJMsb2005114 32202722

